# Accuracy–latency trade-offs and absent embedded validation in deep learning driver drowsiness detection: a PRISMA systematic review

**DOI:** 10.3389/frai.2026.1871926

**Published:** 2026-08-31

**Authors:** Christian Wilbert Salas Yupanqui, Cristhian Edy Llanque Tipo, Frank Diego Choquehuanca Huayhua, Angel Rosendo Condori-Coaquira, David Mamani-Pari, Milton Edward Humpiri-Flores, Esteban Tocto-Cano

**Affiliations:** Escuela Profesional de Ingeniería de Sistemas, Universidad Peruana Unión, Juliaca, Perú

**Keywords:** ADAS, computer vision, deep learning, driver drowsiness, edge computing, PERCLOS, systematic literature review, YOLO

## Abstract

**Introduction:**

Driver drowsiness is a leading cause of road traffic fatalities worldwide, and the convergence of computer vision and deep learning has transformed driver-state monitoring by enabling non-invasive detection within Advanced Driver Assistance Systems (ADAS).

**Methods:**

This systematic literature review, conducted using the PRISMA 2020 methodology and the Kitchenham protocol, synthesizes findings from 33 peer-reviewed studies published between January 2021 and October 2025 to examine the architectures, biomarkers, performance benchmarks, and deployment barriers of deep-learning-based drowsiness detection.

**Results:**

The literature is dominated by convolutional single–pass designs; no reviewed model combines an accuracy above 99% with a latency below 100 ms, and none were evaluated on embedded or automotive–grade hardware.

**Discussion:**

This distribution highlights a persistent accuracy-latency trade-off and emphasizes the need for embedded hardware and cross–dataset evaluation. By synthesizing the available evidence, this review characterizes the current state of the art and proposes a prioritized framework for selecting deep learning architectures for embedded ADAS applications.

## 1 Introduction

Driver drowsiness is a critical andrecurrent contributor to road traffic accidents worldwide, with severe consequences for mortality and socioeconomic costs (Wang, 2025). Estimates attributed to the National Highway Traffic Safety Administration associate driver fatigue with thousands of collisions annually, many of which are fatal or involve serious injury, as summarized in the reviewed literature by Makhmudov et al. (2024). Even transient reductions in alertness can substantially increase crash risk, underscoring the sensitivity of driving performance to fluctuations in the cognitive state (Al-Gburi et al., 2025b). Consequently, Advanced Driver Assistance Systems (ADAS) capable of real-time drowsiness detection constitute an important road-safety intervention rather than merely a technological advancement (Lakshmi Devi and Vinoth, 2025).

Recent advances in computer vision (CV) and deep learning (DL) have substantially advanced driver drowsiness detection by shifting the methodological emphasis toward scalable, non-invasive solutions (Wang, 2025). Earlier approaches relied on physiological signals, such as electroencephalography and electrocardiography, which, although accurate, are often impractical for real-world deployment because of their intrusive nature (Liu et al., 2025). Consequently, contemporary research prioritizes video-based behavioral analysis supported by optimized neural network architectures that enable efficient real-time inference. State-of-the-art models include YOLOv8 and its variants, such as ME-YOLOv8 and YOLOv8-FD, alongside lightweight architectures such as MobileNetV2, which aim to balance detection performance and computational efficiency (Rajput et al., 2025; Li and Hu, 2025). Furthermore, emerging approaches based on Vision Transformers and multimodal frameworks show potential for improving robustness under challenging conditions, including low illumination and partial facial occlusion, thereby supporting model generalizability in realistic driving environments (Cao et al., 2025; El-Nabi et al., 2025).

Despite these advances, the research landscape remains fragmented, particularly in terms of deployment constraints (Lakshmi Devi and Vinoth, 2025). Existing reviews document substantial gains in detection accuracy but often provide limited analysis of the relationship between algorithmic complexity and hardware limitations, which strongly influence real-time feasibility. To date, the literature has not established a standardized evaluative framework that integrates performance metrics with embedded-system constraints in Advanced Driver Assistance Systems (ADAS). Most prior systematic reviews prioritized classification accuracy while giving comparatively less attention to assessments of frames per second relative to predictive performance and deployability under edge-computing conditions. Consequently, a critical knowledge gap persists regarding which high-precision architectures can operate within strict computational budgets without compromising real-time functionality, thereby limiting their practical adoption in industrial applications.

This study addresses these limitations through a systematic literature review (SLR) that synthesizes and critically evaluates studies published between January 2021 and October 2025, with an explicit focus on deployment feasibility in embedded systems. This study makes three primary contributions. First, it classifies state-of-the-art algorithms according to their feasibility and computational requirements. Second, it identifies the structural trade-offs between accuracy and latency that constrain the current detection systems. Third, it proposes a prioritized research agenda to bridge the gap between theoretical performance and practical improvements in road safety. To address these objectives, the review is guided by the following four research questions: (RQ1) Which deep learning architectures are most frequently used for driver drowsiness detection between 2021 and October 2025? (RQ2) Which facial biomarkers and physiological signals are employed in drowsiness monitoring systems, and what levels of reliability do they demonstrate? (RQ3) How do predictive accuracy, latency, frame rate, and hardware requirements interact within the current detection architectures? (RQ4) Which technical and hardware-related factors constrain the real-time deployment of high-accuracy models in embedded Advanced Driver Assistance Systems (ADAS)? The remainder of this paper is organized as follows. Section 2 outlines the methodology, including the review protocol, PRISMA 2020 reporting framework, Kitchenham-based selection and appraisal procedure, and data extraction form. Section 3 presents the findings, organized around six dimensions: architectural prevalence, biomarker reliability, dataset heterogeneity, accuracy–latency trade-off, deployment barriers, and risk of bias together with reporting completeness. Section 4 discusses the theoretical and practical implications of these findings, states the limitations of the review, and concludes in Section 5 with prioritized recommendations and a synthesis framework for selecting deep learning architectures for embedded ADAS environments.

## 2 Methodology

This systematic literature review was conducted in accordance with the Preferred Reporting Items for Systematic Reviews and Meta-Analyses (PRISMA 2020) guidelines and a structured review protocol adapted from Kitchenham and Brereton (2013) to promote transparent and reproducible study identification, selection, and synthesis of studies. The methodology was organized into three sequential phases: (A) search design and study selection, (B) eligibility assessment and quality evaluation, and (C) data extraction and thematic synthesis. Together, these phases establish a systematic and auditable chain from the executed query to each synthesized claim.

Reporting followed PRISMA 2020, and the appraisal procedure followed the review protocol of Kitchenham and Brereton (2013). The review was not registered prospectively, so no timestamped protocol exists. The implications are stated in Section 4.6.

### 2.1 Data sources and search strategy

A comprehensive search was conducted across three major academic databases–Scopus, Web of Science, and Dimensions–selected for their broad coverage of multidisciplinary research, including computer science and engineering. This approach followed the reporting principles of the PRISMA 2020 guidelines to ensure transparency and replicability. The PICOC framework (Population, Intervention, Comparison, Outcome, and Context) was employed to systematically define the scope of the review, as detailed in Table 1.

**Table 1:** Application of the PICOC model in the systematic review.

Component	Description
Population	Drivers monitored through Advanced Driver Assistance Systems (ADAS) in real-world and simulated driving environments.
Intervention	Deep Learning architectures, including Convolutional Neural Networks (CNNs), Transformers, and hybrid models applied to drowsiness detection.
Comparison	Evaluation of different models and algorithms in terms of accuracy, computational efficiency, and deployment feasibility.
Outcome	Detection accuracy, computational efficiency (latency, FPS), robustness, and false positive/negative rates.
Context	Real-world driving environments with real-time constraints and limitations inherent to embedded systems and edge computing.

The search strategy combined Boolean operators (and, or) with a predefined set of keywords derived from the PICOC components. Specifically, the Population component informed the drowsiness-related terms in the first clause, whereas the Intervention component informed the computer vision and deep learning terms in the second and third clauses, respectively. The Outcome component informed the performance-related terms in the fourth clause (*performance, accuracy, detection rate, F1-score, precision*, and *recall*). The Context component, which specifies embedded and edge deployment, was not translated into query terms; the consequences of this omission are analyzed in Section 4.6. The string reproduced below states the Boolean logic common to the three databases. Because query syntax, field tags, and default indexing scope differ across platforms, the string was adapted to the native syntax of each database; the field restrictions applied are stated in the paragraph that follows it. The core search string was:

(“Driver Drowsiness” OR “Driver Fatigue” OR “Driver Sleepiness” OR “Driver State Monitoring” OR “Drowsy Driving” OR “Vigilance”) AND (“Computer Vision” OR “Image Processing” OR “Machine Vision” OR “Video Analysis”) AND (“Deep Learning” OR “Artificial Intelligence” OR “CNN” OR “Convolutional Neural Network” OR “Neural Networks” OR “Face Detection” OR “Facial Analysis” OR “Gaze Tracking” OR “Head Tracking”) AND (“Performance” OR “Accuracy” OR “Detection Rate” OR “F1-Score” OR “Precision” OR “Recall”)

The search was limited to peer-reviewed articles published between 1 January, 2021, and 31 October, 2025, written in English, available through open access, and addressing driver drowsiness detection using computer vision methods. In Scopus and Web of Science, the string was restricted to title, abstract, and keywords (TITLE-ABS-KEY and TS=); in Dimensions, the platform default searches full text. That difference in indexing scope, not in topical coverage, accounts for Dimensions contributing 580 of the 737 records (78.7%) against 44 from Scopus and 113 from Web of Science, and the unqualified term *vigilance* inflated it further. No citation chasing was performed, and no gray literature, preprint servers, or registries were searched. The systematic search was conducted on 31 October 2025, and was not subsequently updated. Consequently, the eligibility window closed on the search date, and articles published after this date were excluded. The interval between the search date and manuscript submission is acknowledged in Section 4.6.

### 2.2 Study selection and eligibility criteria

The study-selection process followed a structured two-stage approach. First, titles and abstracts were screened, followed by a full-text assessment. Both stages were guided by predefined inclusion and exclusion criteria to identify studies that made substantive methodological contributions to the deep learning-based driver drowsiness detection. Table 2 presents the eight inclusion and exclusion criteria applied during the screening process.

**Table 2:** Inclusion and exclusion criteria applied in the systematic review.

Code	Inclusion criteria (IC)	Exclusion criteria (EC)
CI01/CE01	Articles published between 2021 and 2025.	Articles published before 2021 or after 2025.
CI02/CE02	Studies written in English.	Studies published in languages other than English.
CI03/CE03	Research using image or video datasets of drivers as data source.	Studies not using driver images or videos as data source.
CI04/CE04	Works focused on computer science or computer engineering.	Research outside the computational domain (e.g., psychology or sociology without an algorithmic focus).
CI05/CE05	Studies applying computer vision techniques.	Works not employing computer vision methods.
CI06/CE06	Open-access articles with full text available.	Articles without open access or with full text unavailable.
CI07/CE07	Empirical primary studies published in peer-reviewed journals or conference proceedings that report at least one quantitative performance metric obtained from an executed experiment.	Non-empirical works (reviews, surveys, editorials, and position papers), works published without peer review, and works reporting no verifiable quantitative performance metric.
CI08/CE08	Studies focused on driver drowsiness or fatigue detection.	Articles not addressing driver drowsiness or fatigue.

Studies that passed the eligibility screening were subsequently subjected to a structured quality assessment based on eight criteria (Q1–Q8), detailed in Table 3. These criteria evaluated the clarity of the research objectives, contextual description, methodological transparency, experimental rigor, appropriateness of the evaluation metrics, comparative analysis, clarity of the results, and acknowledgment of limitations. Each criterion was scored on a three-point scale (1 = fully satisfied; 0.5 = partially satisfied; 0 = not satisfied), yielding a maximum score of eight points per study. Studies scoring below four points (50% of the maximum score) were excluded from the final analysis. Two reviewers independently assessed each study and resolved disagreements through discussion. Residual disagreements were adjudicated by the corresponding author. The threshold was fixed before extraction and applied at the full-text stage only. Because it is a conventional rather than a validated cut-off, the distribution of scores across the eight criteria and the effect of a stricter cut-off are not reported; Section 3.7 states what the review does and does not report on this point.

**Table 3:** Quality assessment criteria for primary studies.

ID	Assessment question	Purpose
Q1	Are the research objectives or the problem to be addressed clearly defined in the article?	To determine whether the study accurately defines its research objectives and scope.
Q2	Is the context of the study adequately described?	To facilitate understanding of the research setting and application domain.
Q3	Is the proposed methodology or algorithm explained in sufficient detail to allow understanding and potential replication?	To assess the clarity and reproducibility of the methodological approach.
Q4	Is the validation or experimentation process rigorous and methodologically sound?	To identify whether the study employs an adequate and well-founded experimental design.
Q5	Are the evaluation metrics (e.g., accuracy, FPS) appropriate for the problem and clearly justified?	To analyze whether the metrics used are relevant and correctly justified for evaluating model performance.
Q6	Do the authors compare their results with the state of the art or with a baseline method?	To determine whether the study includes meaningful comparative analysis.
Q7	Are the results presented clearly and analyzed in a coherent manner?	To assess the clarity, consistency, and interpretability of the reported findings.
Q8	Does the study discuss its own limitations or threats to the validity of its results?	To identify acknowledged limitations and potential directions for future work.

Records retrieved from the databases were processed using the PARSIFAL platform. Duplicate entries were automatically removed, followed by manual screening of the titles, abstracts, and full texts. Both reviewers screened all records independently at every stage.

The selection process unfolded across the four sequential phases prescribed by PRISMA 2020:

- **Identification:** The database search retrieved 737 records (Scopus: 44; Web of Science: 113; Dimensions: 580). Automatic duplicate detection in Parsifal removed 122 duplicate records, leaving 615 unique records remaining.
- **Screening:** Manual screening of titles, abstracts, and introductions reduced the initial pool from 615 records to 57 studies retained for full-text review; 558 records were excluded at this stage.
- **Eligibility:** the 57 studies were assessed in full text against the eight inclusion and exclusion criteria (CI01–CE08, Table 2) and the minimum quality threshold (Q1–Q8, Table 3). This process excluded 24 studies on five grounds, each mapped to the criterion it invokes: (i) absence of a verifiably reported quantitative performance metric (CI07/CE07); (ii) methodological description insufficient to support critical appraisal (Q3–Q4); (iii) substantive thematic and methodological redundancy with an already included record; (iv) unavailability of the full text through open access (CI06/CE06); and (v) scope outside the computer-science domain or absence of an algorithmic approach to driver drowsiness detection (CI04/CE04, CI08/CE08).
  Redundancy was defined as the simultaneous presence of three conditions: the records used the same algorithmic pipeline, evaluated it on the same dataset or datasets, and reported performance values differing by less than one percentage point. Retaining both records under these conditions would have duplicated the same evidence without contributing distinct findings to the synthesis. Where the two independent reviewers disagreed on whether a record pair satisfied these conditions, both records were retained provisionally, and the disagreement was adjudicated by the corresponding author.
- **Inclusion:** A total of 33 studies satisfied all eligibility and quality criteria and were incorporated into the final analysis presented in Section 3.

**Figure 1:**
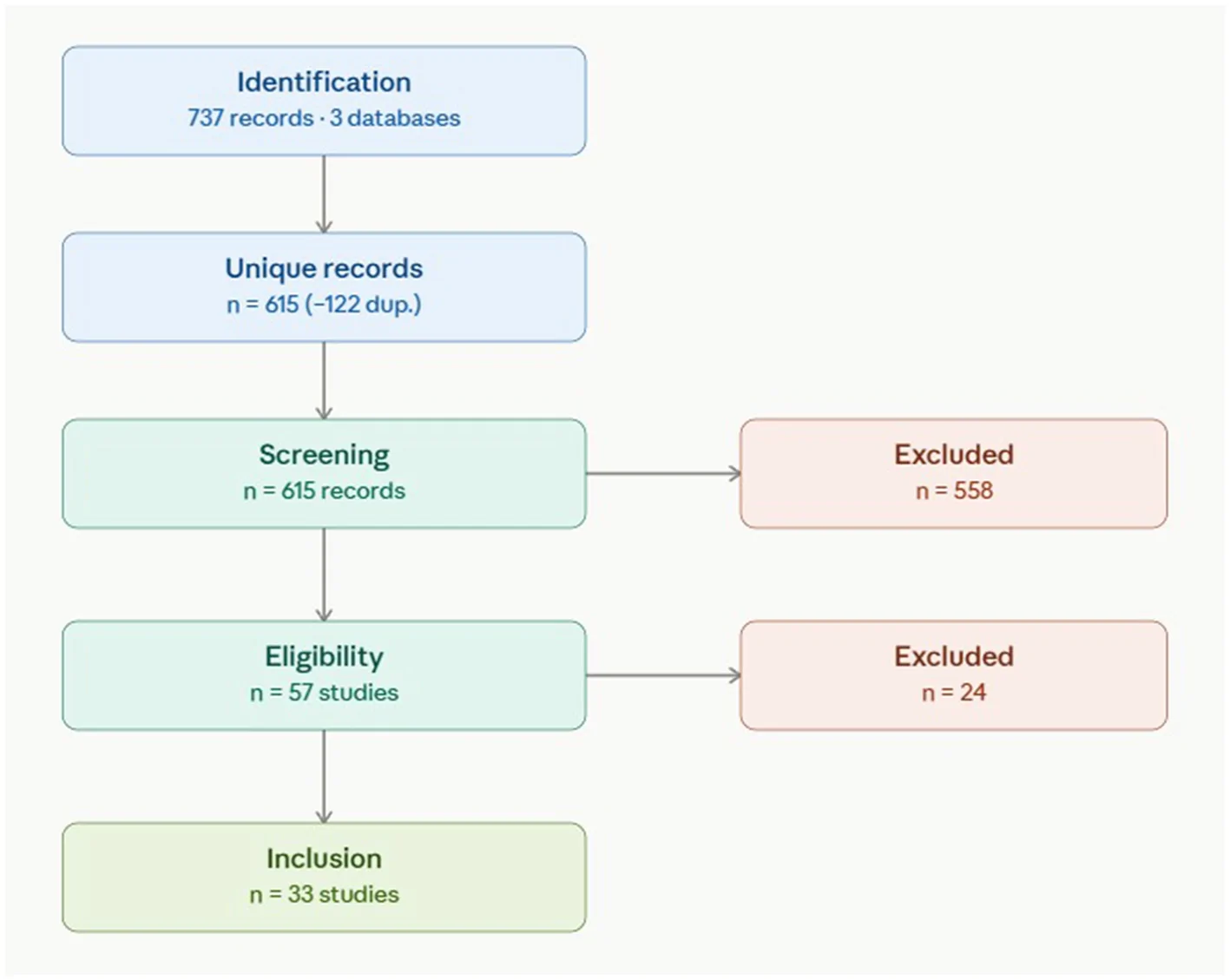
**Identification:** 737 records retrieved from three databases (Scopus: 44; Web of Science: 113; Dimensions: 580); after removing 122 duplicate records, 615 unique records remained. **Screening:** of the 615 records assessed by title and abstract, 558 were excluded and 57 advanced to the eligibility phase. **Eligibility:** the 57 studies were assessed at full text; 24 were excluded for failing criteria CI01–CE08 (Table 2) or the minimum quality threshold Q1–Q8 (Table 3). **Inclusion:** 33 studies met all criteria and were incorporated into the final analysis Section 3).

The PRISMA 2020 framework guided the study-selection process and supported a structured and traceable representation of filtering across these four stages, as illustrated in Figure 1.

### 2.3 Data extraction

Data were extracted independently and in duplicate for all 33 studies into a form fixed before the full-text stage, covering 14 variables: primary architectural family and components present, modality, biomarkers, dataset and its provenance, sample size, task type, performance metric as labeled by the source study and its value, latency, frame rate, parameter count or floating-point operations, evaluation hardware, and acknowledged limitations. Discrepancies were resolved by discussion, with the corresponding author adjudicating.

Two coding rules govern how the tables should be read. Assignment to a primary architectural family followed the contribution declared in each source study's title, not the reviewers' judgment of which component dominated computation; component prevalence (the *All* column of Table 4) was coded separately and non-exclusively. Unreported values were coded as *not reported* and never inferred; therefore, the completeness figures in Section 3.7 measure the literature rather than the extraction. Heterogeneity in datasets, hardware, task types, and metric definitions precluded pooling; therefore, no synthesis beyond frequency counts and tabulation was attempted.

**Table 4:** Distribution of architectural families used in driver drowsiness detection (2021–October 2025; *N* = 33 studies).

Architecture	Type	Modality	Primary	All	References
YOLO family (v5, v8, FD, and FDCL)	CNN/hybrid	Visual	9	9	Liu et al., 2025; Lakshmi Devi and Vinoth, 2025; Rehman et al., 2025; Li and Hu, 2025; Essahraui et al., 2025; Alemdar and Codur, 2024; Debsi et al., 2024; Sim and Kim, 2024; Ran et al., 2023
Standard and pretrained CNNs (ResNet, VGG, MobileNet, ShuffleNet, and EfficientNet)	CNN	Visual	10	10	Wang, 2025; Rajput et al., 2025; Badri et al., 2025; Chew et al., 2024; Yang and Yi, 2024; Venkateswarlu and Ch, 2024; Makhmudov et al., 2024; Jahan et al., 2023; Al-Gburi et al., 2025a; Bhanja et al., 2025
Deep Neural Networks (DNN)	CNN	Physiological	2	3	Yang and Yi, 2024; Ko et al., 2025; Al-Gburi et al., 2025b
Temporal Transformers (TFT, Swin Transformer)	Transformer	Multimodal/Visual	3	3	Assarzadeh et al. (2025); El-Nabi et al. (2025); Xu et al. (2025)
CNN-Transformer hybrids	Hybrid	Multimodal/Visual	5	5	Zhang et al., 2025; Jiao et al., 2025; Phan et al., 2025; Venkateswarlu and Venkata Rami Reddy, 2025; Gaikwad et al., 2023
Sequential hybrids (CNN + LSTM/RNN/MLP/ANN)	Hybrid	Multimodal/Visual	3	5	Venkateswarlu and Venkata Rami Reddy, 2025; Zaman et al., 2025; Venkateswarlu and Ch, 2024; Jasim et al., 2022; Cao et al., 2025
Classical machine learning (kernel SVM)	Non-DL	Visual	1	1	Ahirwar and Kushwah, 2021

CNN, convolutional neural network; DNN, deep neural network; TFT, temporal fusion transformer; SVM, support vector machine.

*Count columns. Primary* is mutually exclusive and sums to 33. *All* measures component prevalence and sums to 36, the difference arising from three studies that span two families: Venkateswarlu and Ch (2024), Venkateswarlu and Venkata Rami Reddy (2025) and Yang and Yi (2024). The percentages in the text are derived from *Primary*; *Reference* lists every study counted under *All*. *Correction*. Ahirwar and Kushwah (2021), previously counted under *Temporal Transformers*, proposed a kernel SVM rather than an attention-based architecture and is reported here as a separate classical category; those counts are therefore three, not four, and the row totals are unchanged.

### 2.4 Thematic synthesis

The synthesis was organized thematically to capture the principal applications of deep learning in driver drowsiness detection. The findings were categorized into five application areas: YOLO-based architectures, standard and pre-trained convolutional neural networks, CNN-Transformer hybrid models, sequential hybrid architectures, and physiological-signal models. These categories were analyzed alongside cross-cutting dimensions, including the accuracy–latency trade-off, deployment barriers, literature and knowledge gaps, trust and user acceptance, methodological capabilities, and future research directions. This thematic organization provides a structured overview of the state of the art represented in the retrieved corpus and the challenges associated with the transition from laboratory research to operational deployment in vehicles.

## 3 Results

Based on the synthesis of the 33 selected studies, the literature shows a progression from static feature extraction to temporal and hybrid modeling, organized around three dominant design paradigms: (i) lightweight real-time detectors based on YOLO architectures, (ii) high-precision spatiotemporal CNN-Transformer hybrids, and (iii) computationally efficient visual–physiological frameworks. Rather than devoting a separate subsection to each paradigm, the results are organized around six cross-cutting dimensions derived from the research questions: architectural prevalence, biomarker reliability, dataset heterogeneity, accuracy–latency trade-off, deployment barriers, and risk of bias together with reporting completeness. Therefore, each subsection examines how the three paradigms perform within the corresponding dimension, and the cross-sectional synthesis at the end of the section integrates these findings. Two conventions apply throughout the paper. Percentages are computed on the mutually exclusive *primary* classification of Table 4 unless stated otherwise, and every quantitative claim states the denominator on which it rests, because reporting is incomplete and uneven across the corpus (Section 3.7).

### 3.1 Architectural dominance

The systematic analysis indicates that driver drowsiness detection is dominated by convolutional architectures, particularly single-stage detectors and standard or pre-trained convolutional neural networks, accompanied by an increasing use of global attention mechanisms, as summarized in Table 4 (Liu et al., 2025; Alemdar and Codur, 2024; Debsi et al., 2024). Within the reviewed corpus, this distribution reflects a consistent prioritization of real-time performance. Two architectural families account for most of the evidence in comparable proportions: single-stage YOLO detectors (v5, v8, FD, and FDCL), which constitute the primary contribution of nine of the 33 studies (27.3%) and balance detection accuracy with inference speed (Lakshmi Devi and Vinoth, 2025; Alemdar and Codur, 2024); and standard or pre-trained convolutional neural network backbones, such as ResNet, VGG, MobileNet, and EfficientNet, which constitute the primary contribution of 10 studies (30.3%) (Bhanja et al., 2025; Al-Gburi et al., 2025a). These families overlap conceptually because YOLO detectors are convolutional, and several backbone-based studies use these networks as feature extractors within detection pipelines. Their combined prevalence indicates that the corpus is dominated by convolutional and single-pass inference rather than attention-intensive or two-stage designs. Hybrid CNN-Transformer models represent a principal area of architectural innovation, constituting the primary contribution of five studies (15.2%) (Zhang et al., 2025; Jiao et al., 2025; Venkateswarlu and Venkata Rami Reddy, 2025). Sequential hybrids that combine convolutional feature extraction with recurrent or fully connected temporal modeling constitute the primary contribution of three studies (9.1%) and appear as components in five studies overall (Cao et al., 2025; Zaman et al., 2025). Specialized deep neural networks for physiological signal processing and temporal Transformer architectures maintained a smaller but functionally significant presence, representing the primary contributions of two (6.1%) and three studies (9.1%), respectively (Assarzadeh et al., 2025; Al-Gburi et al., 2025b). Unless stated otherwise, these percentages are derived from the mutually exclusive primary classification reported in Table 4 and therefore sum to 100%. The same table also reports component prevalence, under which a study that incorporates two architectural families is counted in both categories. One further study (Ahirwar and Kushwah, 2021) contributed a kernel support vector machine rather than a deep architecture (1 study; 3.0%). It was retained because it satisfied the eligibility criteria as applied at the full-text stage, but it was reported as a separate classical-machine-learning category rather than absorbed into any deep learning family; the implications of its presence for the scope of the corpus are stated in Section 4.6. Both dominant families are organized around inference cost rather than accuracy alone: single-stage detectors avoid the region-proposal stage of two-stage pipelines, and lightweight backbones reduce multiply–accumulate operations through depthwise separable or channel-shuffled convolutions, in both cases trading representational capacity for video-rate inference on constrained hardware. Two design philosophies are recurrent. Low- to medium-complexity architectures, including YOLO detectors and lightweight CNNs such as ShuffleNet, prioritize efficiency and are candidates for embedded deployment (Liu et al., 2025; Yang and Yi, 2024); High-complexity hybrid and multimodal models use cross-attention to fuse facial features with electroencephalography and electrooculography (Ko et al., 2025; Jiao et al., 2025), extending representational capacity at a higher computational cost (Ran et al., 2023; Al-Gburi et al., 2025a).

### 3.2 Biomarkers and indicators

Recent research (2021–October 2025) shows increasing interest in multimodal driver drowsiness detection systems that integrate behavioral and physiological indicators to reduce diagnostic uncertainty, as summarized in Table 5 (Liu et al., 2025). This development extends the field from single-feature visual approaches to the fusion of facial metrics with electroencephalography, electrooculography, and electrocardiography signals. Such integration can yield an accuracy above 95% in some experimental settings by compensating for modality-specific limitations under variable environmental conditions (Cao et al., 2025; Al-Gburi et al., 2025a). Within the reviewed corpus, visual biomarkers remain predominant because they support non-invasive monitoring, with the Percentage of Eye Closure, Eye Aspect Ratio, and Mouth. The aspect Ratio is widely used in the literature (Makhmudov et al., 2024; Sim and Kim, 2024). However, physiological signals contribute to multimodal detection. Electroencephalography can support the detection of cognitive fatigue before observable behavioral manifestations, whereas electrooculography-based analysis of slow eye movements has achieved an accuracy of up to 99.89% under controlled conditions (Zhang et al., 2025; Jiao et al., 2025). Despite these advantages, visual biomarkers can exhibit relative performance reductions exceeding 40% under low illumination or facial occlusion, highlighting their sensitivity to real-world driving conditions (Zhang et al., 2025). Conversely, physiological indicators may provide complementary information through the direct measurement of neurological, ocular, or cardiac activity, but their deployment remains constrained by sensor intrusiveness, hardware requirements, and user discomfort, which limit their scalability in practical applications (Bhanja et al., 2025; Liu et al., 2025).

**Table 5:** Biomarkers used in driver drowsiness detection.

Biomarker	Type	Associated architectures	Advantages/limitations	Evidence (studies)
PERCLOS (Percentage of Eyelid Closure)	Visual	CNN, YOLOv5, VGG16, and ResNet50	Advantage: Gold standard for visual fatigue detection. Limitation: Sensitive to low-light conditions and eyewear.	Makhmudov et al., 2024; Liu et al., 2025; Ran et al., 2023
EAR (Eye Aspect Ratio)	Visual	MediaPipe, YOLOv8, and DrowsyDetectNet	Advantage: Fast processing suitable for real-time operation. Limitation: Error increases with significant head rotations.	Essahraui et al., 2025; Badri et al., 2025; Venkateswarlu and Ch, 2024
MAR/Yawn detection	Visual	YOLOv5 + Swin Transformer, ME-YOLOv8	Advantage: Identifies advanced fatigue and drowsiness states. Limitation: May be confused with speech-related movements.	Li and Hu, 2025; Debsi et al., 2024; Makhmudov et al., 2024
Head pose (Euler angles and nodding)	Visual	Attention Mesh 3D, CDIDM (time series)	Advantage: Detects microsleeps and prolonged inattention. Limitation: Requires high resolution to maintain accuracy.	Ran et al., 2023; Sim and Kim, 2024
EEG (Alpha, Beta, and Theta bands)	Physiological	SLEEP-SAFE (CNN), TMU-Net (Transformer)	Advantage: Early detection of cognitive fatigue. Limitation: Intrusive due to electrode contact.	Ko et al., 2025; Zhang et al., 2025; Al-Gburi et al., 2025b
EOG/SEM (Slow Eye Movements)	Physiological	PMMCT (CNN-Transformer), TMU-Net	Advantage: More stable than EEG against physiological noise. Limitation: Requires specialized hardware.	Jiao et al., 2025; Zhang et al., 2025
Heart rate/rPPG	Physiological	Dual-Modal CNN (ResNet, DenseNet)	Advantage: Non-invasive when obtained via camera (rPPG). Limitation: Sensitive to motion and lighting variations.	Chew et al., 2024

PERCLOS, Percentage of Eyelid Closure; EAR, Eye Aspect Ratio; MAR, Mouth Aspect Ratio; EEG, Electroencephalography; EOG, Electrooculography; SEM, Slow Eye Movements; rPPG, remote photoplethography.

Evidence column. Studies from which each row's advantages and limitations were extracted, so that no claim in the table is unsourced; the Associated Architectures column is descriptive, not exhaustive.

### 3.3 Dataset heterogeneity

The 33 included studies used highly heterogeneous datasets for training, validation, and testing. These datasets range from widely used public benchmarks, such as YawDD and NTHU-DDD, to author-constructed datasets, often collected under laboratory conditions that vary substantially in terms of the completeness of their reporting across studies.

Table 6 summarizes the datasets used across the 33 included studies, aggregated by dataset and modality.

**Table 6:** Frequency of use of public datasets across the 33 included studies.

Dataset	No. of studies (%)	Modality
YawDD/YAWDD	9 (27.3%)	Visual (facial video)
NTHU-DDD	7 (21.2%)	Visual (facial video, simulated conditions)
MRL eye dataset	5 (15.2%)	Visual (eye image)
DROZY	3 (9.1%)	Multimodal (visual + physiological)
UTA-RLDD	3 (9.1%)	Visual (facial video)
SEED-VIG	3 (9.1%)	Physiological (EEG)
CEW (closed eyes in the wild)	2 (6.1%)	Visual (eye image)
DrivFace	1 (3.0%)	Visual (facial video)

A single study may use more than one dataset (cross-dataset validation); therefore, the so percentages do not sum to 100%. In addition to the public datasets listed, at least 11 of the 33 studies (33.3%) relied, wholly or partly, on in-house or non-standardized datasets collected by the authors.

This distribution reveals two important patterns: First, no public dataset is used by a majority of the studies. The most frequently used dataset, YawDD, appeared in only nine of the 33 studies (27.3%), whereas NTHU-DDD appeared in seven studies (21.2%), indicating substantial fragmentation in the experimental benchmarks of the field; only seven studies (21.2%) evaluate on two or more of these public benchmarks. Second, at least 11 of the 33 studies (33.3%) relied wholly or partly on in-house, non-standardized datasets whose acquisition conditions, including illumination, participant number and demographic diversity, and camera type, were often insufficiently documented to support independent replication. This combination of benchmark fragmentation and limited standardization hinders direct comparison across architectures and may reduce the external validity of the aggregate conclusions presented in Section 3.4. An architecture reporting superior accuracy may benefit from a less demanding dataset, such as one with more favorable illumination or lower participant diversity, rather than an inherent algorithmic advantage. Therefore, future research should adopt cross-dataset validation protocols that include multiple public datasets and explicitly report the demographic and environmental characteristics of each dataset. These practices would allow the generalization capacity of each architecture to be assessed more independently of the dataset-specific characteristics.

### 3.4 Accuracy–latency trade-off

The analysis of the technical feasibility of Advanced Driver Assistance Systems indicates a consistent pattern, as summarized in Table 7: lightweight and re-parameterized architectures generally achieve a more favorable balance between predictive accuracy and inference latency than computationally intensive models. Real-time vehicular systems impose strict latency constraints, but the corpus supports statements about them for only a minority of the included studies. Table 7 reports the ten models for which a timing measurement or a parameter count could be extracted: nine of the 33 studies (27.3%) reported a timing measurement of any kind, and only seven (21.2%) reported it as a per-frame latency. Therefore, every claim in this subsection is conditioned on those denominators rather than the full corpus. Among the models reporting both accuracy and per-frame latency, three combine accuracy above 95% with latency below 100 ms: ME-YOLOv8 (97.5%, 12.2 ms), YOLOv8-FD (98.06%, 16.7 ms), and EffRes-DrowsyNet (98.07%, 28.0 ms) (Debsi et al., 2024; Lakshmi Devi and Vinoth, 2025; Al-Gburi et al., 2025a). Two of these models are single-stage YOLO detectors, whereas the third is a compact convolutional hybrid, suggestting that computationally efficient designs are currently best positioned to satisfy both performance constraints simultaneously. Models with lower floating-point operation counts, such as TMU-Net (94.3%, 9.4 ms) and the FNCA-based network (94.29%, 10.4 ms per sample) (Zhang et al., 2025; Al-Gburi et al., 2025b), achieved comparable or lower latency but remained below the 95% accuracy threshold. By applying the viability rule stated in the caption of Table 7 (accuracy ≥94% and per-frame latency ≤ 33 ms), the four models classified as highly viable span 94.3%–98.07% accuracy at 9.4–28.0 ms, corresponding to a latency-derived throughput of approximately 36–106 frames per second. The two thresholds are reported separately because they are not interchangeable, and throughput and end-to-end latency are retained as distinct measures: converting a reported frame rate into a latency presupposes unbatched and non-pipelined inference, an assumption the none of the reviewed studies states explicitly (Debsi et al., 2024; Al-Gburi et al., 2025a). Architectures such as ME-YOLOv8 use Efficient Channel Attention mechanisms to improve feature selection while maintaining a low inference latency, whereas TMU-Net attains an inference time of 9.4 ms despite incorporating uncertainty quantification (Zhang et al., 2025). In contrast, computationally intensive models, including EfficientNetB0 and Swin Transformer variants with diffusion-based denoising, achieve an accuracy approaching 99.9% but incur inference latencies exceeding 1.0 s per frame (Wang, 2025; El-Nabi et al., 2025). This latency substantially exceeds the approximately 33 ms per-frame budget required for operation at 30 fps, which limits their suitability for real-time embedded ADAS deployment without substantial architectural optimization.

**Table 7:** Accuracy–latency trade-off in drowsiness detection models for ADAS deployment.

Model	Task/metric	Accuracy (%)	Latency (ms)	As reported	Hardware (context)	ADAS viability
ME-YOLOv8	Det./acc.	97.5	12.2	82 FPS	NVIDIA RTX 4060, 8 GB (desktop GPU)	High (desktop GPU)
YOLOv8-FD	Det./acc.	98.06	16.7	60 FPS	Intel i7 11th Gen/RTX 3050 (desktop GPU)	High (desktop GPU)
TMU-Net	Class./acc.	94.3	9.4	9.4 ms	NVIDIA RTX 4090 (workstation GPU)	High (workstation GPU)
EffRes-DrowsyNet	Class./acc.	98.07	28.0	35–40 FPS/28 ms	Quadro M4000/Xeon E5 (workstation GPU)	High (workstation GPU)
FNCA + DNN	Class./acc.	94.29	n/a^‡^	10.4 ms/sample	Intel i7-10750H, CPU only (laptop CPU)	Not determinable
YOLO-FDCL	Det./acc.	98.8	n/r^*^	—	Intel i9-13900H/RTX 4070 (desktop GPU)	Not determinable
DLID3-ADAS (ShuffleNet)	Class./acc.	97.05	600	0.60 s/frame	Intel i5-8600K/GTX 1050 Ti (desktop GPU)	Low
DrowsyDetectNet	Class./acc.	99.23	164.4	164.4 ms	Intel i5 13th Gen, 8 GB RAM (desktop CPU)	Medium (desktop CPU)
EfficientNetB0	Class./acc.	99.9	n/a^‡^	1.06 s/sample	NVIDIA RTX 3070 (desktop GPU)	Not determinable
Swin Transformer + diffusion denoising	Class./acc.	99.94	860–1830	0.86–1.83 s/frame	Not reported^†^	Low

^*^Parameters reported (10.96 M), but no timing measurement.

^†^Inference time reported without identifying the platform.

^‡^Reported per sample, not per frame; the span of a sample is unspecified; therefore, no equivalent latency (n/a) or viability class was assigned. All other latencies are per frame; n/r, not reported.

*Conversion*. Frame rates were converted as 1, 000/FPS; seconds were converted to milliseconds; and where both were reported, the stated latency was used.

*Viability. High*: accuracy ≥94% and latency ≤ 33 ms.; *Medium*: accuracy ≥94% and 33 < latency ≤ 500 ms.; *Low*: latency >500 ms.; *Not determinable*: no comparable per-frame measurement.

*Caveat*. The latency depends on the platform and is not comparable across rows. No model was evaluated on embedded or automotive-grade hardware; therefore, every class is provisional. The laptop-CPU result of FNCA + DNN is the strongestindirect evidence of resource-efficient execution in the corpus. FPS, frames per second.

*Task and metric. Det*., detection; *Class*., frame- or segment-level classification; *acc*., accuracy, as labeled by the source study. All 10 report accuracy, including those whose native task is detection and for which mean average precision would be conventional; the two are not commensurable, so this column gives within-study indicators, not a common scale. *Throughput vs. Latency*. The figures converted from a reported frame rate assume unbatched, non-pipelined inference and are therefore upper-bound throughput estimates, not response times. No study stated whether its timing included face detection and post-processing; therefore, all values were recorded as reported. *Missing columns*. The parameter counts, GFLOPs, memory footprint, and power consumption are not tabulated because fewer than a third of the studies reported them (Section 3.7).

### 3.5 Feasibility and deployment barriers

The deployment of driver drowsiness detection systems in real-world environments is constrained by computational, memory, and thermal limitations, as summarized in Table 8. Architectures based on Vision Transformers and diffusion models, despite achieving high predictive performance under laboratory conditions, may be difficult to deploy on low-power embedded hardware because of their computational complexity and memory requirements (El-Nabi et al., 2025; Al-Gburi et al., 2025b; Liu et al., 2025; Lakshmi Devi and Vinoth, 2025). This discrepancy between benchmark performance and operational feasibility remains a central unresolved challenge, limiting the translation of state-of-the-art models into practical Advanced Driver Assistance Systems. Table 8 enumerates the five barrier categories identified by the corpus, together with the affected architectures and reported mitigations, and is not restated here. Two of them are quantified: diffusion-based inference at 0.86–1.83 s per frame exceeds the 33 ms budget associated with 30 frames per second by a factor of 26–55, and purely visual pipelines lose more than 40% of the relative accuracy under low illumination or facial occlusion. The proposed mitigations are structural rather than architectural post-training quantization, structured pruning, and reparameterization- and none have been validated on a target device (Wang, 2025).

**Table 8:** Main technical barriers and deployment feasibility of drowsiness detection models in ADAS systems.

Limitation	Affected architectures	Impact on ADAS	Reported mitigation strategies
High computational complexity (static demand: parameter count, GFLOPs, and memory footprint)	Transformers (ViT, Swin), diffusion models, and deep CNNs (VGG19, ResNet152)	High VRAM and static power consumption; overheating and reduced stability in embedded systems. Independent of execution time: a model may exceed the memory budget of a target device even if it would run fast enough on it	Lightweight backbones (MobileNetV4, ShuffleNet), block reduction, and fusion layer simplification
Inference latency (dynamic demand: execution time under load)	Iterative diffusion models and complex hybrid architectures	0.86–1.83 s per frame; incompatible with the 25–30 FPS real-time threshold. Distinct from complexity: a compact model may still be slow if its computation is inherently sequential, as in iterative denoising	Quantization, model pruning, TensorRT and TFLite optimization
Environmental sensitivity	Purely visual models (EAR/MAR), standard CNNs	Relative accuracy reductions above 40% reported under low illumination (Liu et al., 2025) and partial facial occlusion (Zhang et al., 2025); *n* = 2 studies with non-comparable baselines, datasets, and operational definitions of the adverse condition	rPPG integration, CLAHE illumination normalization, and infrared sensors
Intrusiveness and user rejection	Physiological systems based on EEG and EOG with wet electrodes	Physical discomfort and low driver acceptance in real-world scenarios	Discrete behind-the-ear sensors, wireless headbands, non-invasive multimodal fusion
Scarcity of labeled data	All deep learning architectures	Overfitting risk and poor generalization across ethnicities, ages, and real-world conditions	Self-supervised learning, pretext tasks, and synthetic data augmentation via GANs

### 3.6 Cross-sectional synthesis

Table 9 integrates the four research questions and separates three deployment profiles: high viability (selected YOLO models and non-contact visual–rPPG systems, real-time under the reported conditions but environmentally sensitive), medium viability (deep CNNs, temporal Transformers and CNN-Transformer hybrids, accurate under controlled conditions but limited by memory demand and sequential bottlenecks), and low viability (contact-based physiological models, fast and accurate but constrained by intrusiveness). No reviewed architecture satisfies all four evaluative dimensions simultaneously; therefore, the accuracy–latency–deployability trade-off remains unresolved. Two reading conditions apply: the performance ranges aggregate within-study values obtained on different datasets, tasks and platforms and are not estimates of a population parameter; and the number of supporting studies is stated per row, with single-study rows marked as such, because a range derived from one observation conveys a spurious impression of variability.

**Table 9:** Cross-sectional synthesis of architectures, biomarkers, performance, and deployment feasibility in ADAS (RQ1–RQ4).

Architectural domain (RQ1)	Dominant biomarkers (RQ2)	Typical performance (RQ3)	Main barriers (RQ4)	ADAS deployability	Supporting studies
YOLO family (v5, v8, FD, and FDCL)	PERCLOS, EAR, and MAR	97%–99% 60–82 FPS	Sensitivity to light and facial occlusion	High	*n* = 9; performance from *n* = 2 (Debsi et al., 2024; Lakshmi Devi and Vinoth, 2025)
Lightweight CNNs (MobileNet, ShuffleNet, and EfficientNet-B0)	EAR, MAR	94%–99% 30–45 FPS	Accuracy drop in nighttime scenarios	High/medium	*n* = 10; performance from *n* = 3 (Yang and Yi, 2024; Venkateswarlu and Ch, 2024; Wang, 2025)
Temporal Transformers (ViT, Swin, and TFT)	PERCLOS, Head Pose, EEG/EOG	94%–98% High compute cost	Dependence on high-end hardware	Medium	*n* = 3 (Assarzadeh et al., 2025; El-Nabi et al., 2025; Xu et al., 2025)
CNN-Transformer hybrids	Visual + physiological (multimodal)	96%–99% Variable FPS	Fusion complexity and memory footprint	Medium	*n* = 5 (Zhang et al., 2025; Jiao et al., 2025; Phan et al., 2025; Venkateswarlu and Venkata Rami Reddy, 2025; Gaikwad et al., 2023)
Sequential hybrids (CNN + LSTM/RNN)	PERCLOS, Head Pose (temporal)	95%–97% Moderate latency	Slow sequential processing	Medium	*n* = 5 (Venkateswarlu and Venkata Rami Reddy, 2025; Zaman et al., 2025; Venkateswarlu and Ch, 2024; Jasim et al., 2022; Cao et al., 2025)
Pure physiological models (EEG, EOG)	EEG, EOG, SEM	94%–99% Low latency	Intrusiveness and low user acceptance	Low	*n* = 3 (Ko et al., 2025; Al-Gburi et al., 2025b; Zhang et al., 2025)
Non-invasive multimodal (Vision + rPPG)	PERCLOS + HR/rPPG	96%–98% (single study) Near real-time	Sensitivity to motion and light	High	*n* = 1 (Chew et al., 2024)

Reading this table. The *Supporting studies* column reports the number of included studies assigned to each architectural domain and, where the performance range derives from a smaller subset, the studies from which it was obtained. Ranges aggregate within-study values obtained on heterogeneous datasets, tasks, and platforms and are not pooled estimates; rows supported by a single study are marked as such and must not be read as ranges.

### 3.7 Risk of bias and reporting completeness

Every included study scored at or above the 4/8 appraisal threshold, which follows from the eligibility rule rather than constituting an independent finding.

Reporting completeness (Table 10) was the principal source of bias in this review, and its direction was known. Because the search string required an accuracy-related term (Section 2.1), the corpus over-represents studies that foreground accuracy: accuracy is reported universally, timing by fewer than a third of the studies, and memory footprint, power consumption and embedded evaluation by none. Therefore, every quantitative claim in Sections 3.4 and 3.6 is conditioned on a reporting subset rather than on the corpus.

**Table 10:** Reporting completeness across the 33 included studies.

Reported item	Studies, *n* (%)	Derived from
Quantitative accuracy or an equivalent performance metric	33 (100.0)	Criterion CI07
A timing measurement of any kind (latency, frame rate or per-sample time)	9 (27.3)	Table 7
A per-frame latency, convertible to a real-time budget	7 (21.2)	Table 7
The evaluation platform, alongside a timing or complexity measurement	9 (27.3)	Table 7
Memory footprint or power consumption	0 (0.0)	Table 7
Two or more of the public datasets listed in Table 6	7 (21.2)	Section 3.3
At least one author-constructed dataset	11 (33.3)	Table 6
Evaluation under an explicitly adverse condition against a baseline	2 (6.1)	Section 4.2
Evaluation on embedded or automotive-grade hardware	0 (0.0)	Section 4.5

Every value is derived from the source stated in the third column and can be recomputed from it. The table reports only items that the present synthesis extracted; it is not a complete inventory of what the primary studies disclosed. The percentages were computed for the full corpus of 33 studies.

One gap concerns the outcome declared in Table 1, which includes false positive and false negative rates. Accuracy is the only performance metric extracted for any of the models in Table 7, and no false-negative rate appears in the reviewed evidence. Because alert frames vastly outnumber drowsy frames in every dataset used, a model can attain high aggregate accuracy while missing most drowsiness episodes, which is a failure mode with safety consequences. Therefore, the corpus does not permit the assessment of the failure mode for any reviewed model, which is a limitation of the evidence base rather than the synthesis.

## 4 Discussion

This systematic review synthesizes 33 empirical studies on deep learning-based driver drowsiness detection (2021–October 2025). It classifies the reviewed architectures by their reported real-time feasibility and computational requirements, characterizes the empirical boundary between accuracy and latency in the corpus, and derives a prioritized research agenda. Each of these is developed below in relation to the corresponding research questions.

### 4.1 Architectural dominance and its practical implications (RQ1)

Standard or pre-trained convolutional backbones (30.3% of the included studies) and single-stage YOLO detectors (27.3%) accounted for most of the reviewed evidence, indicating a consistent prioritization of single-pass inference pipelines that minimize latency while preserving detection accuracy. The central finding is not which family ranks first. The primary classification in Table 4 is mutually exclusive, so the two counts are directly comparable; they nonetheless differ by one study, or three percentage points, on a corpus of 33, and a Wilson interval for that difference comfortably includes zero. Therefore, no claims of rank are made. What the distribution shows is that attention-intensive and two-stage designs remain a minority throughout, a pattern that differs from pre-2021 reviews reporting the predominance of two-stage detectors and conventional convolutional neural networks (CNNs) (Sikander and Anwar, 2019; Ngxande et al., 2020).

The emergence of CNN-Transformer hybrids (15.2%) indicates an increasing interest in architectures capable of modeling spatiotemporal dependencies. Their contributions are not incrementally accurate. Drowsiness manifests through temporal combinations of events—prolonged eye closure, repeated yawning, and progressive head movement—rather than through any single frame, and self-attention provides a direct computational path between distant positions in a sequence where convolutions require depth to do so, at a memory cost that grows with sequence length. The second contributions are attention-based fusion: the cross-attention mechanisms in Jiao et al. (2025) and Zhang et al. (2025) allow the weight of each modality to vary with signal quality, converting multimodality from redundancy into a potential source of robustness. Neither contribution has been isolated from other architectural components in any of the reviewed studies.

Physiological-signal-based models (6.1% as a primary contribution) occupy a limited but important position at the accuracy frontier, with a reported accuracy of at least 99.89% when electrooculography is integrated (Jiao et al., 2025). Their practical adoption remains constrained by contact-based sensing requirements, hardware complexity, and user discomfort. This divergence between the maximal predictive performance and deployability highlights a structural tension that architectural refinement alone may not resolve.

### 4.2 Biomarker reliability and multimodal complementarity (RQ2)

Percentage of Eye Closure (PERCLOS), commonly defined as the proportion of frames in which the eye aperture falls below 20% of its maximum within a one-minute interval, remains the most extensively validated visual biomarker in the wider literature and is represented across the reviewed corpus (Table 5). The review did not tabulate biomarker reporting frequency by study, so no claim about its relative frequency within the corpus is made here, in the abstract, or in Section 5; establishing such a claim would require adding a biomarker-frequency field to the extraction form and recoding the 33 studies, which is recommended for the next iteration of this review. What the corpus does supports a statement about robustness under adverse conditions, which is developed below. Two studies evaluated visual detection under explicitly adverse conditions, and both reported relative accuracy reductions exceeding 40%: YOLO-FDCL (Liu et al., 2025) under low-illumination footage and TMU-Net (Zhang et al., 2025) under partial facial occlusion. Their baselines, datasets, and operational definitions of the adverse condition differ; therefore, the figure indicates the approximate magnitude of the problem rather than a pooled effect. The practical consequence is that a model reporting an accuracy near 99% on a curated dataset such as CEW or UTA-RLDD cannot be assumed to retain it under low illumination, occlusion, or non-frontal head orientation. Accuracy must therefore be read together with the dataset and acquisition conditions that produced it; the influence of computing hardware is treated separately in Section 4.4, and the fragmentation of datasets across the corpus in Section 3. That no other included study reported a controlled comparison against a baseline condition, which is itself a reporting gap worth naming.

Multimodal approaches that integrate remote photoplethysmography-derived heart rate with visual features report accuracies between 96% and 98% at near-real-time speeds, suggesting a viable pathway for maintaining non-contact monitoring while increasing physiological specificity. In contrast, electroencephalography-based systems may support the earlier detection of cognitive fatigue (Zhang et al., 2025), but their contact-based sensing requirements limit practical vehicular deployment. Two responses to this constraint are conceivable, and they concede different things. Remote photoplethysmography does not require additional hardware and estimates a pulse from color variations in the facial skin captured by the existing camera (Chew et al., 2024), but its sensitivity to motion and illumination reproduces the environmental limitations it is meant to offset. The alternative relocates contact to components the driver already touches, or to discreet wearables, preserving signal quality at the cost of an additional device. Only the first route appears in the corpus; therefore, the intrusiveness barrier is widely acknowledged but rarely addressed by construction.

### 4.3 The accuracy–latency trade-off: a structural boundary (RQ3)

Among the models reporting both magnitudes (Table 7), none simultaneously achieves accuracy above 99% and latency below 100 ms. Two qualifications delimit the claim. It is descriptive rather than optimal—10 measurements on heterogeneous datasets, tasks, and platforms, not strictly commensurable—and, therefore, an empirical boundary of the corpus, not a Pareto frontier. And it rests entirely on desktop and workstation hardware (RTX 3070–4090, a Quadro M4000, desktop-class Intel processors); therefore, on low-power devices, the separation between high-accuracy and real-time models is likely to widen rather than narrow, although only embedded evaluation can establish by how much. High-accuracy models such as EfficientNetB0 (99.9%) and a Swin Transformer with diffusion-based denoising (99.94%) (El-Nabi et al., 2025) operate at 0.86–1.83 s per frame, exceeding the approximately 33 ms budget associated with 30 frames per second by a factor of approximately 26–55, whereas ME-YOLOv8 (97.5%, 82 frames per second) and YOLOv8-FD (98.06%, 60 frames per second) (Debsi et al., 2024; Lakshmi Devi and Vinoth, 2025) achieve substantially lower latency with comparatively small reductions in reported accuracy.

The third qualification concerns the threshold itself. The 33 ms figure is derived from the convention of capturing video at 30 frames per second, not from the detection task: PERCLOS is computed over an extended window, and drowsiness onset unfolds over seconds to minutes; therefore, neither requires that every frame be processed independently as it arrives. Temporal subsampling, event-triggered inference, or batching within the PERCLOS window could meet the same objective at a fraction of the cost; however, the reviewed studies rarely evaluate these alternatives and even more rarely still justify the per-frame assumption they adopt. Making that assumption explicit is itself a finding: part of what the field treats as a hardware limitation may be an artifact of an inherited processing model, and relaxing it is a cheaper route to embedded feasibility than further architectural compression.

The fourth concerns the outcome variables. The boundary is expressed in accuracy because accuracy is the only metric every included study reported, but accuracy is neither commensurable across the detection and classification tasks combined in Table 7 nor operationally adequate under a class imbalance. No study reports a false-negative rate (Section 3.7), so the boundary is a boundary on the metric the field reports, and its practical significance depends on the evidence the field does not yet produce.

This trade-off also reflects the distinct architectural sources of computational demand. Diffusion-based models require iterative refinement, which introduces sequential inference steps, whereas transformer-based models impose substantial computation and memory costs through global self-attention. Although self-attention supports parallel computation, its resource requirements restrict deployment at high spatial and temporal resolutions. Quantization and structured pruning have been reported to reduce latency by 10%–40% without proportional accuracy loss (Zhang et al., 2025), but these gains depend on the architecture, hardware platform, and optimization procedure, and none have been validated on automotive-grade, safety-compliant hardware.

### 4.4 Deployment barriers: beyond computational cost (RQ4)

Deployment barriers extend beyond the accuracy–latency trade-off to include systemic limitations in the evaluation practice. First, the predominant reliance on high-performance academic graphics processing units means that reported metrics cannot be transferred directly to embedded automotive platforms, which operate under stricter memory, energy, and thermal constraints; production systems commonly rely on low-power processors or automotive system-on-chip platforms on which inference latency may increase substantially, and future studies should report latency, memory use, and power consumption on such named targets rather than on desktop devices. Second, the limited use of cross-dataset validation, combined with the reliance on controlled datasets such as CEW, NTHU-DDD, and UTA-RLDD, suggesting that the reported accuracy may overestimate real-world performance (Al-Gburi et al., 2025a). Third, most reviewed studies evaluate detection models in open-loop settings, which do not capture the feedback dynamics of operational ADAS, in which alerts or interventions alter driver behavior and, therefore, subsequent sensor inputs. Fourth, the metrics reported are not those a safety case would require: accuracy is universal, whereas sensitivity, specificity, and false-negative rates are effectively absent (Section 3.7), and memory footprint and power consumption are reported by no included study. A deployment decision requires all four, and the absence of the last three is a barrier to evaluation practice rather than of technology.

Collectively, these limitations indicate that the effective performance of current models in real-world deployments may be lower than benchmark conditions suggest. Addressing this discrepancy requires integrated evaluation frameworks that incorporate target-hardware constraints, environmental variability, and closed-loop system dynamics.

### 4.5 Positioning relative to hardware-based ADAS implementations

Hardware-centric ADAS implementations provide a useful point of comparison. The FPGA-based drowsiness detection and autonomous stopping system reported in Almomany et al. (2025) implements face and eye detection on a Xilinx platform using the Vivado HLS toolchain, combining Viola–Jones feature extraction, pretrained OpenCV classifiers, and a compact CNN for driving-control decisions, and closes the control loop by initiating longitudinal and lateral vehicle control when the driver does not respond to an alert within approximately 4.4 s.

Two differences require emphasis here. The first concerns scope: that study develops and validates a single end-to-end prototype on dedicated reconfigurable hardware, whereas the present review synthesizes 33 studies to characterize field-level trends and the empirical accuracy–latency boundary. The contributions are complementary rather than competing, and the comparison clarifies the purpose of this review: it does not propose an implementation but identifies the conditions under which such implementations are feasible.

The second difference concerns the evaluation substrate and carries a greater methodological weight. None of the 33 included studies reported deployment on an FPGA or automotive-grade reconfigurable hardware, and the frame rates in Table 7 were obtained almost exclusively on desktop graphics processing units. Implementations such as Almomany et al. (2025) demonstrate that low-latency, closed-loop detection is attainable when the pipeline is co-designed with the target device. However, they achieve this with classical or lightweight detectors rather than with the transformer- and diffusion-based architectures that define the reported accuracy frontier. Whether these architectures retain their accuracy after quantization and mapping onto embedded fabrics is an open question that this review can frame but not resolve, and it is carried forward as the first item of the agenda in Section 5.

### 4.6 Limitations of this review

The restriction to open-access publications introduces availability bias by excluding subscription-based and industry evidence relevant to real-world deployment; the temporal scope (2021–October 2025) limits comparison with earlier developments; and the focus on English-language studies may underrepresent demographically distinct research contexts (Al-Gburi et al., 2025a). Together, these filters mean that the architectural frequencies in Section 3 characterize the retrieved corpus rather than the field: they are evidence of what the accessible literature emphasizes, not a census of research or industrial practice.

A further and more consequential limitation concerns the search strings. The PICOC framework in Table 1 defines the context of this review as embedded systems and edge computing; however, the Boolean query in Section 2.1 contains no deployment-oriented term: its fourth clause requires an accuracy-related metric, and no clause admits *embedded, edge, FPGA, latency, inference time*, or *real-time* as an entry point. Studies reporting hardware implementation and timing behavior without foregrounding predictive accuracy were therefore less likely to be retrieved. The bias this introduces is asymmetric, and its direction was known: work validated on embedded hardware is systematically less likely to appear in this corpus than work that is not, so the true landscape probably contains more viable embedded solutions than the corpus displays. The observation that no included study validates on FPGA or automotive-grade hardware should accordingly be read as a gap in the accuracy-reporting literature specifically—a narrower and better-supported claim—and the hardware-oriented implementation discussed in Section 4.5 is itself an example of eligible work absent from the corpus. Replication with deployment-oriented terms would establish how much of the reported gap survives a search designed to find it.

Three additional boundaries apply. First, the review inherits the convention that real-time operation means per-frame processing at 30 fps and uses it to separate viability classes. As argued in Section 4.3, that convention is not derived from the physiology of drowsiness onset, so the resulting classification is conditional on an assumption the field makes rather than on an independently justified requirement. Second, the search was conducted on October 31, 2025, and not updated; the architectural distribution and position of the accuracy–latency boundary are the findings most exposed to this, and a living-review format is the appropriate remedy. Third, the review does not address the ethical and privacy dimensions of driver monitoring—consent, on-device vs. remote processing, retention, secondary use, and demographic fairness—which fell outside the PICOC framework but are not peripheral to deployment: a system that is technically viable and socially unacceptable will not be deployed.

Four limitations concern the conduct of the review rather than the corpus. One included study (Ahirwar and Kushwah, 2021) proposed a kernel support vector machine rather than a deep architecture; it was reclassified accordingly (Table 4), but its retention shows that the boundary between deep learning and classical machine learning was not operationalized sharply enough at screening. Criterion CI07 originally read *high-impact peer-reviewed journals* without a threshold, and no indexing or quartile requirement was enforced. It has been restated in Table 2 to describe what was actually applied. No backward or forward citation chasing was performed, which would have been the least costly partial remedy for the search bias described above. Three quantities that a fully specified review would report are absent here and are named rather than left to be noticed: inter-rater agreement was not quantified, so the consistency of the screening decisions cannot be assessed externally; the number of records attributable to each ground for exclusion is not reported; and the distribution of appraisal scores across the eight criteria is not reported, so the effect of a stricter cut-off cannot be examined. The review was also not registered prospectively, so no external means exists of verifying that the questions, criteria, and analysis plan were fixed before extraction. The documented procedures in Section 2.2 mitigate but do not replace registration, which the authors will undertake in subsequent reviews. Finally, heterogeneity in datasets, hardware, reporting practices, and metrics precluded formal meta-analysis.

## 5 Conclusions

This systematic review synthesizes evidence from 33 peer-reviewed studies (2021–October 2025) to characterize the state of the art in deep learning-based driver drowsiness detection across four dimensions: architectural dominance, biomarker reliability, the accuracy–latency trade-off, and embedded deployment feasibility. One caveat conditions every quantitative statement below and is not repeated thereafter: all reviewed measurements were obtained from desktop or workstation hardware, and none on an embedded or automotive-grade platform. Therefore, the findings describe the accessible literature rather than verified in-vehicle performance.

Convolutional single-pass designs dominate the corpus of the study. Standard or pre-trained CNN backbones appeared in 10 studies (30.3%) and single-stage YOLO detectors in 9 (27.3%), whereas attention-intensive and two-stage designs remained a minority. Only two of the nine YOLO studies reported any timing measurements; both reach 60–82 frames per second at 97%–98% accuracy (Debsi et al., 2024; Lakshmi Devi and Vinoth, 2025). The real-time suitability of the family as a whole is therefore suggested by two cases rather than established, and the deficit is one of reporting practice rather than of demonstrated performance. CNN-Transformer hybrids (five studies; 15.2%) define the current innovation frontier through spatiotemporal modeling, but no reviewed studies validates them on edge hardware.

PERCLOS is the most extensively validated visual biomarker in the wider literature and is represented throughout the corpus, although its reporting frequency was not tabulated, and no claim of relative frequency was made. The two studies that evaluated visual detection under explicitly adverse conditions reported relative accuracy reductions above 40% under low illumination and partial facial occlusion (Liu et al., 2025; Zhang et al., 2025); because their baselines and definitions differ, this indicates substantial environmental sensitivity rather than a general upper bound. The fragility originates in the underlying eye-aperture estimate rather than in the metric, and per-driver adaptive thresholding, illumination normalization, infrared imaging, and fusion with head pose are stabilization strategies the corpus does not yet compare. Electrooculography-derived metrics have reached 99.89% accuracy (Jiao et al., 2025) but require contact-based sensing.

Only nine of the 33 studies (27.3%) reported a timing measurement of any kind and only seven (21.2%) reported a per-frame latency, among these, none simultaneously exceeded 99% accuracy and 100 ms latency. This is an empirical boundary of what the corpus reported rather than a formal Pareto frontier, since the underlying accuracies were obtained from heterogeneous datasets, tasks, and platforms and are not strictly commensurable. ME-YOLOv8 (≥97% at 12.2 ms), YOLOv8-FD (98.06% at 16.7 ms), and EffRes-DrowsyNet (98.07% at 28.0 ms) offered the most favorable reported trade-off, whereas transformer architectures with diffusion denoising exceeded the 33 ms threshold by one to two orders of magnitude. No study reports a false-negative rate; therefore, none of these figures speaks to the failure mode that matters for road safety. For practitioners choosing an architecture today, the evidence favors lightweight single-stage detectors as a default, subject to verification on the intended target device. Table 8 lists the five persistent structural barriers.

The following six research directions are proposed. (i) Optimization techniques should be validated on named embedded or automotive-grade platforms, reporting latency, memory use, and power consumption alongside predictive performance. (ii) The field requires standardized, publicly released multimodal datasets spanning illumination, weather, occlusion, vehicle type, and driver demographics, and cross-dataset validation should become routine. (iii) Non-contact sensing, such as—remote photoplethysmography, thermal imaging, and sensors integrated into vehicle components,—should be developed with user acceptance measured empirically. (iv) The operational definition of *real time* should be derived from the temporal dynamics of fatigue rather than from the inherited 30 frames-per-second convention, with adaptive sampling and event-triggered inference assessed as alternatives. (v) The ethical, legal, and privacy dimensions of driver monitoring require on-device biometric processing, explicit retention policies, and demographic fairness audits. (vi) Reporting practice should change before architectures do: studies should report sensitivity, specificity, and false-negative rates alongside accuracy, state the task and the native metric, and specify whether reported timings include face detection and post-processing. Without these, no accuracy figure in this literature can support a deployment decision.

These conclusions are limited by the restriction to open-access English-language sources, temporal scope, and omission of deployment-oriented terms from the search query. This omission biases the corpus against hardware-validated work the absence of which this review reports. Replicating the review with such terms, and broader language coverage, is the condition under which its central claim can be confirmed or overturned.

## Data Availability

The original contributions presented in the study are included in the article/supplementary material, further inquiries can be directed to the corresponding author/s.
